# Comparison of three fixation methods for orotracheal intubation in 95 adults

**DOI:** 10.1186/s40001-020-00446-x

**Published:** 2020-10-02

**Authors:** Ye Sun, Hua Fan, Xiao-Xia Song, Hua Zhang

**Affiliations:** Emergency Department of China-Japan Friendship Hospital, No.2 of Yinghua East Street, Chaoyang District, Bejing, 100029 China

**Keywords:** Endotracheal intubation, Holder, Teeth pad, Critical illness, Intensive care unit (ICU)

## Abstract

**Background:**

The present study aimed to compare three fixation methods for orotracheal intubation.

**Methods:**

Through literature retrieval, the effects of the adhesive/twill tape method, fixator method, and adhesive/twill tape–fixator alternation method on patients with tracheal intubation in the intensive care unit (ICU) were compared.

**Results:**

The fixator and alternation methods were more effective in protecting the tongue mucosa and teeth. The alternation method was superior to the other two methods in maintaining the position of the endotracheal intubation. However, the difference in facial and lip injuries between the three methods was not statistically significant.

**Conclusion:**

The fixator method can significantly reduce intraoral injury and is more suitable for older people with weak tongue mucosa and loose teeth. These are worth popularizing among a wider group.

## Background

Endotracheal intubation refers to the technique of inserting a tracheal catheter through the mouth or nasal cavity, and through the glottis into the trachea. It provides the best conditions for airway patency, oxygen supply, respiratory tract suction, and anti-aspiration and plays an important role in the rescue of critical patients [1]. However, since the application of this technique, there is no unified method for the fixation of tracheal intubation, and complications caused by the fixation, such as facial, lip, and oral mucosal damage, and catheter displacement and tooth loosening, are common. Once complications occur, these can induce airway mucosal damage, tracheal spasm, prolonged hospitalization, and increased infection opportunities and mortality [2]. Traditional studies are limited to the empirical use of fixation methods, lack assessment of the specific situation of intubated patients, and lack assessment tools. In the present study, more than 100 studies at home and abroad were reviewed, and 43 articles on the renewal of fixation materials and improvement of methods for adult orotracheal intubation in the previous 4 years were summarized [3–8]. Then, 95 emergency intensive care unit (EICU) patients with tracheal intubation were divided into three groups for a trial using the improved and innovative method recommended by the literature, and the advantages and disadvantages of different fixation methods were identified. In addition, the related factors in patients with complications were also identified. The rates of catheter displacement and skin breakage were decreased by the innovative fixation method.

## Materials and methods

### Subjects

The present study is a case–control study; patients who were admitted to the EICU from January 2018 to July 2019 were enrolled. According to the order of tracheal intubation after hospitalization, these patients were randomly divided into three groups and observed using the inter-group control method. Three methods were used to fix the tracheal intubation of patients: method 1, the twill tape method; method 2, the fixator method; and method 3, the alternation method. The present study was approved by the Ethics Committee of our hospital, and all patients signed informed consent.

### Inclusion and exclusion criteria

Inclusion criteria: (1) patients admitted to the EICU who needed orotracheal intubation, and (2) patients who were > 18 years old. Exclusion criteria: (1) patients with oral and maxillofacial injuries; (2) patients with a history of allergies to the experimental materials; (3) patients with facial skin infections, such as furuncle and carbuncle; and (4) patients who received fewer than 6 days of tracheal intubation.

### Operation method

EICU nurses who had worked for more than 5 years participated in the present study, and the researchers provided uniform training for the participants. All nurses were trained in the three methods of endotracheal intubation fixation, and the use of experimental materials and oral nursing.

**Fig. 1:**
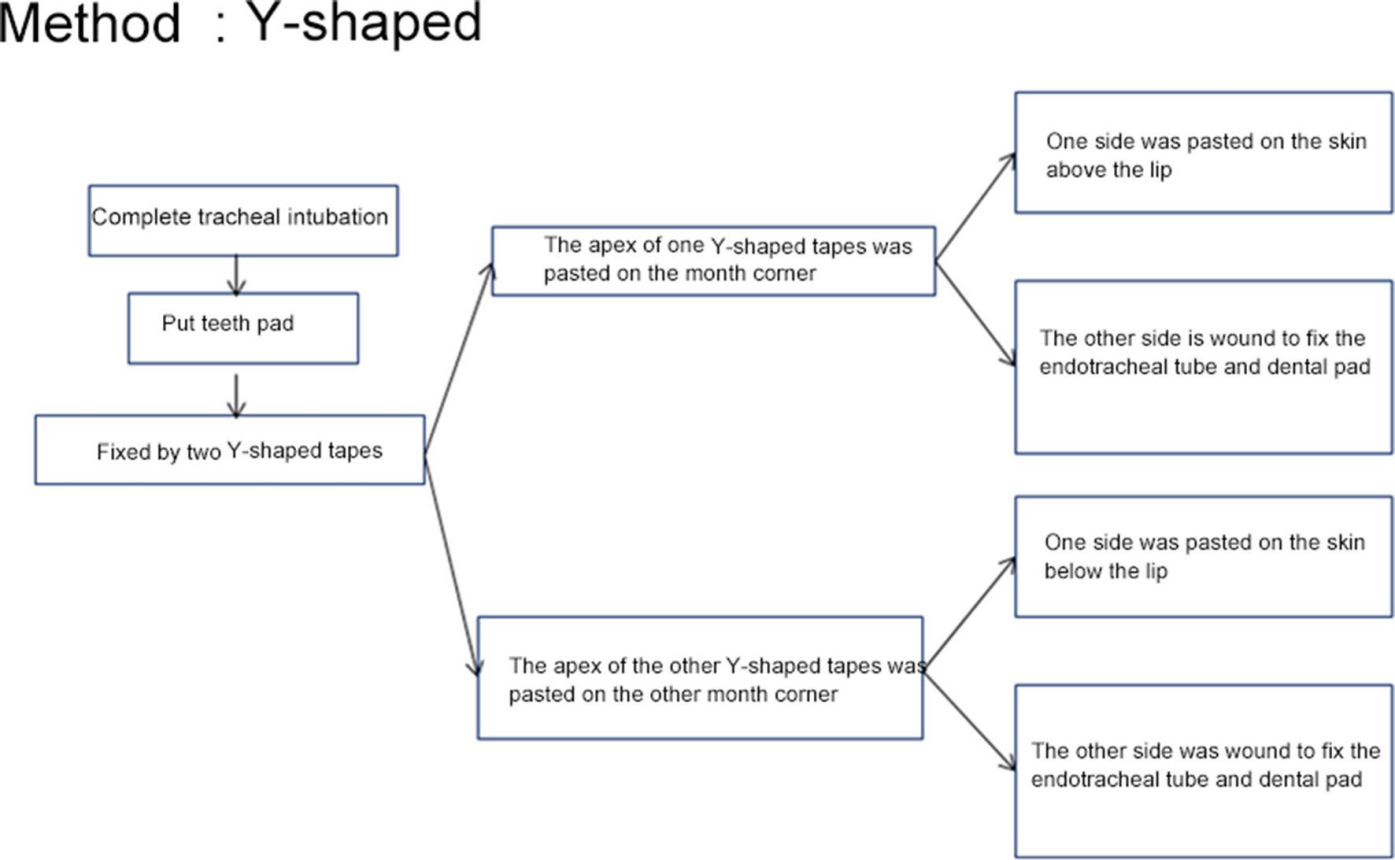
Adhesive tape and twill tape fixation: "Y" shape

**Fig. 2:**
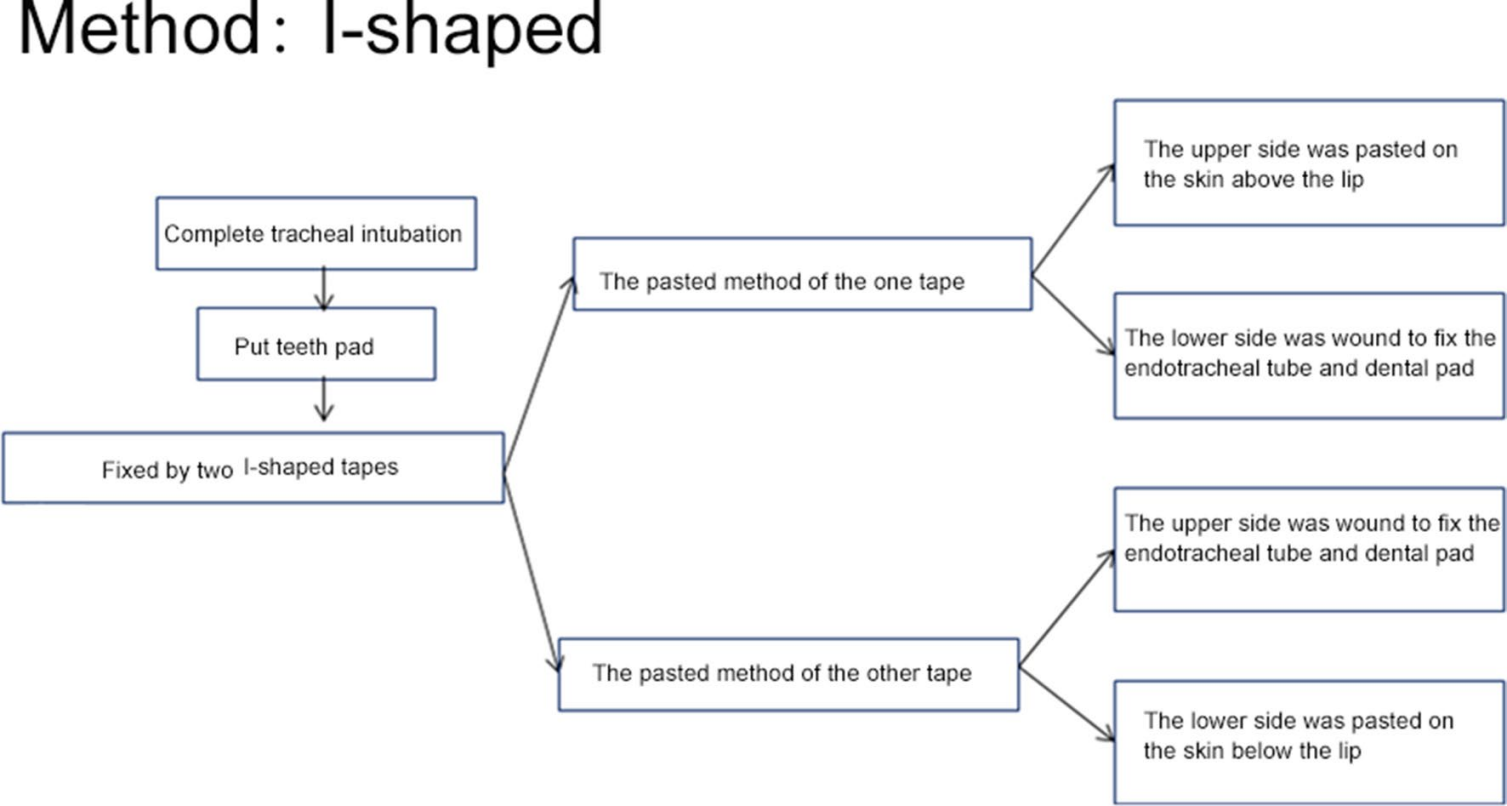
Adhesive tape and twill tape fixation: "I" shape

**Fig. 3:**
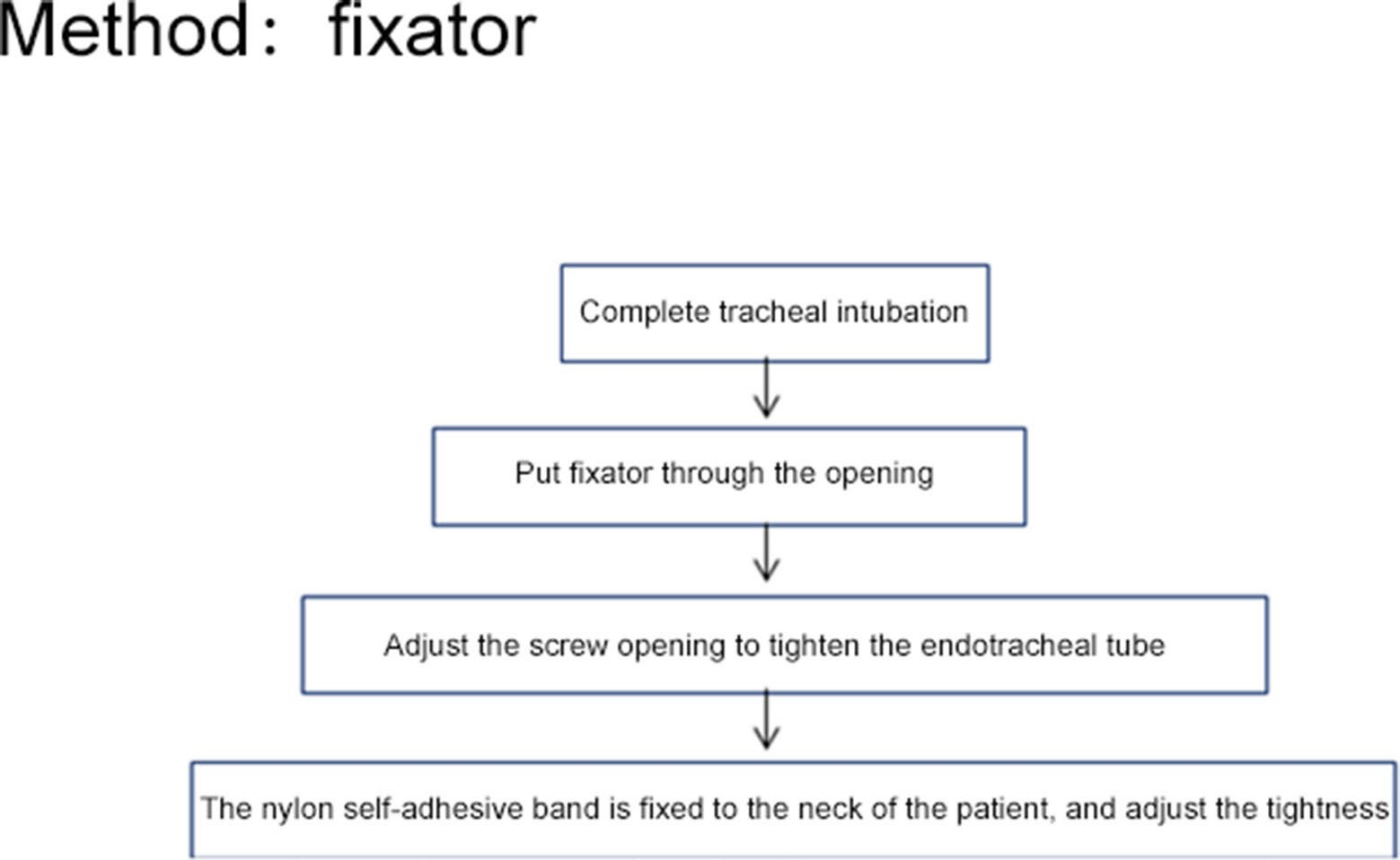
Fixator method

Adhesive tape and twill tape fixation: tapes were cut into the "Y" and "I" shape (Figs. 1, 2, 3). After successful intubation, a mouth pad (Model 40; Yangzhou Guilong Medical Instrument Co., Ltd.) was placed and fixed with two Y-shaped tapes. Preparation of the Y-tape: a 10 × 2 cm rectangular tape was cut from the middle of the short side into a "Y" shape, and the two sides were 8 cm long. The top of the first tape was stuck to the corner of the mouth. One side was stuck to the skin above the lip, and the other side was wound around and fixed to the intubation catheter and mouth pad. The top of the second tape was stuck to the contralateral corner of the mouth, and the entangling method was the same as the first tape. The nurses removed the adhesive tape, twill tape, and mouth pad every day for oral care. After oral care, the tracheal intubation was fixed using the same method. The damaged skin was steered when the adhesive tape was applied.

A "Walker" disposable bite block was used. Device features: it is a plastic product, which is shuttle-shaped, 11 cm long and 6 cm wide, and designed according to the radian of the human face and oral cavity. The inner layer has a decompression soft film to reduce the pressure on the facial skin. In the middle, it has an opening for the tracheal intubation. The tightness of the intubation fixation can be adjusted by a screw knob on one side. A 2-cm-long and 1-cm-wide bite block is attached to the intubation catheter, and there is a heart-shaped opening on the other side to suck the sputum. The device is disposable. After successful intubation, the catheter was inserted from above or below the patient’s lips through an opening to fix the intubation catheter. After the intubation was fixed, the device was entangled and fixed to the patient’s neck using self-adhesive nylon tapes, which are 16 cm or 36 cm long and 2 cm wide on both sides of the device. After oral care, the tracheal intubation was fixed with the same method.

**Fig. 4:**
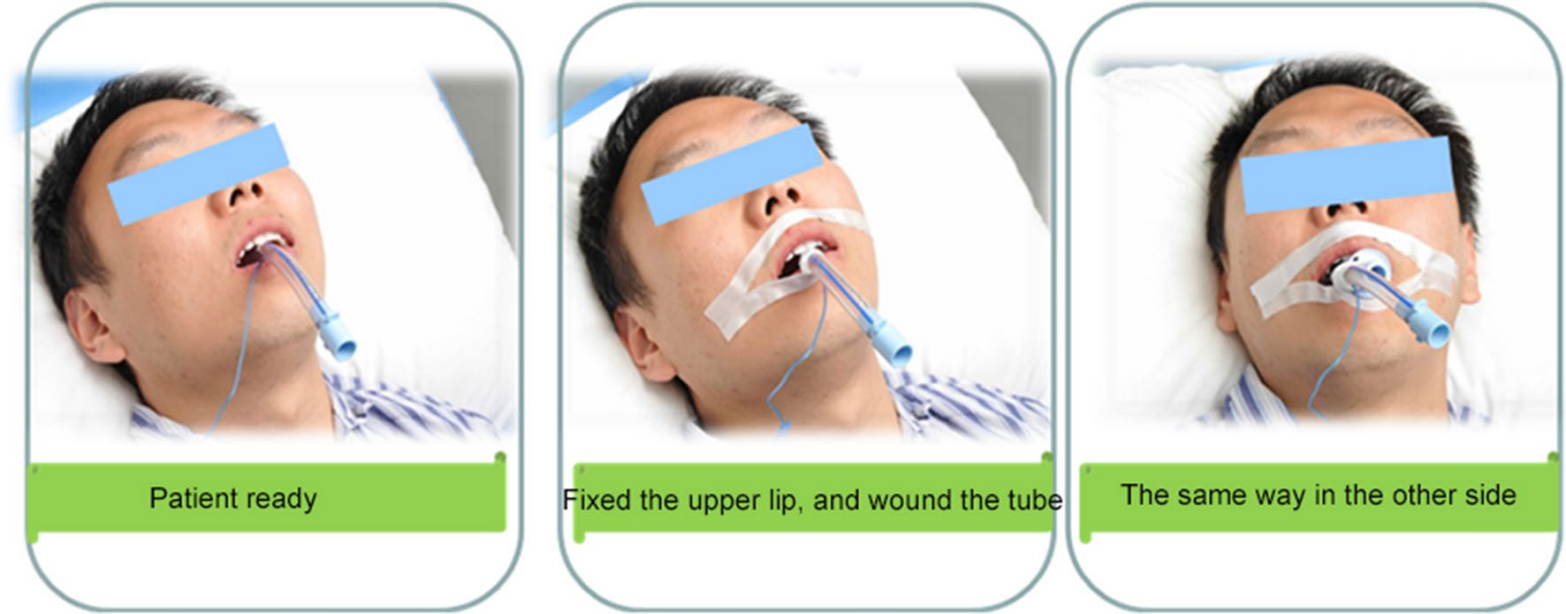
Specific operational process

**Fig. 5:**
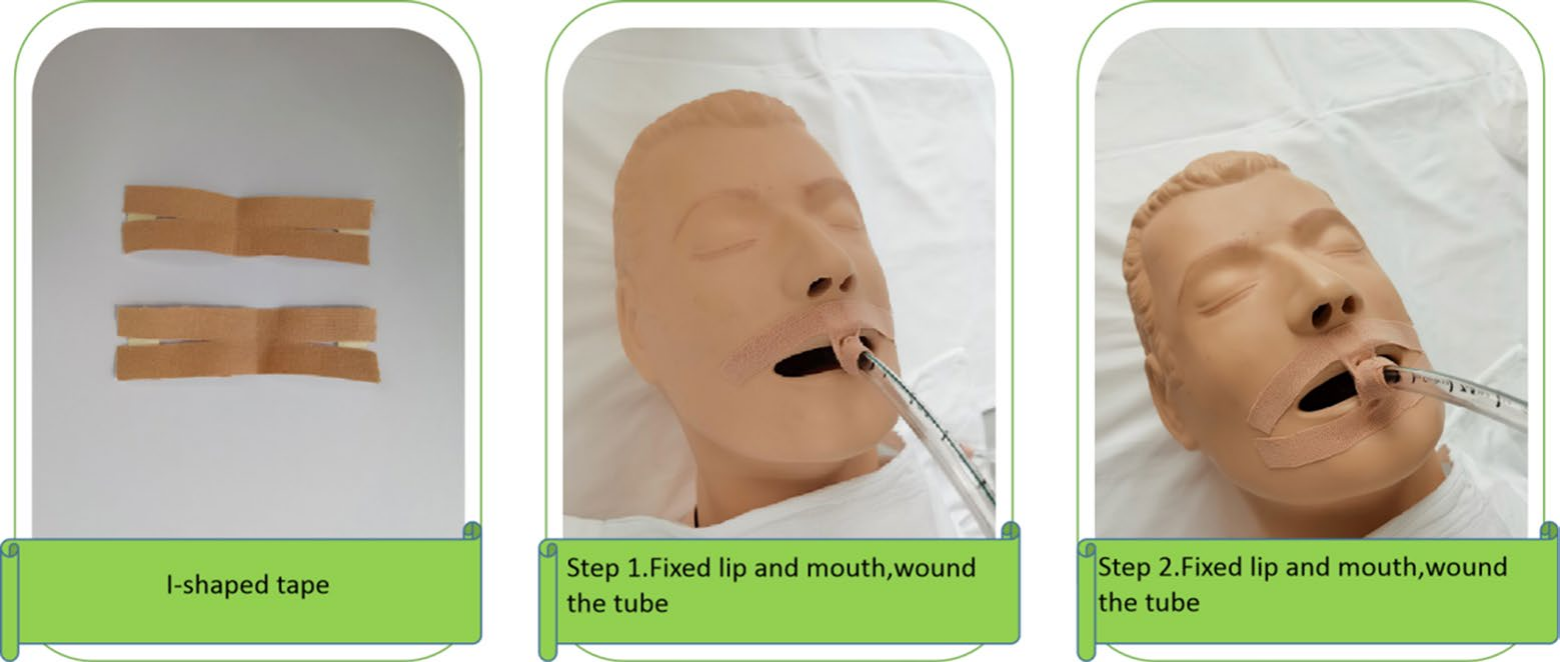
Tear the tape into "I" shape

**Fig. 6:**
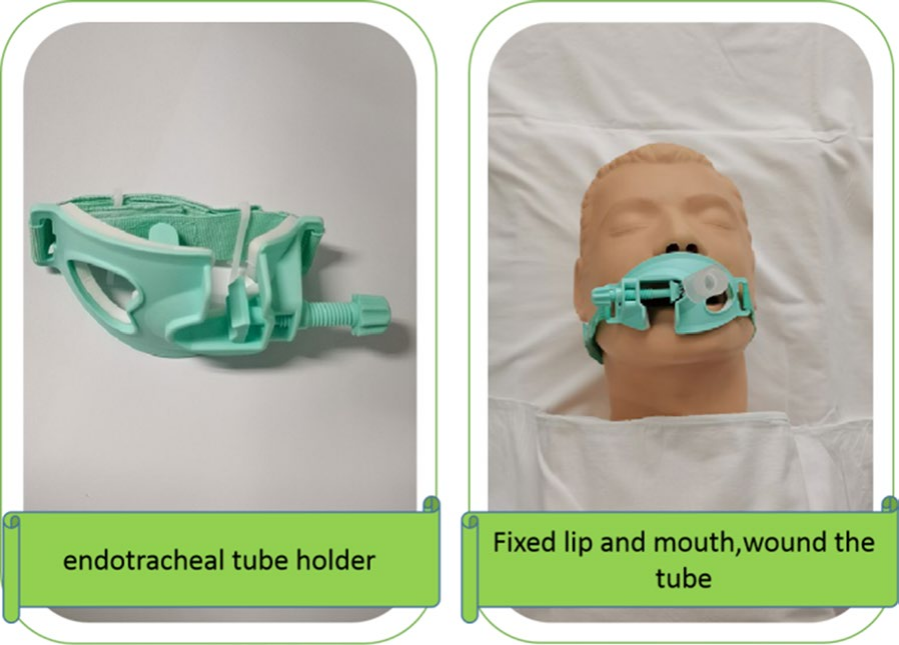
"Walker" disposable bite block

Alternation method: the fixator and twill tape methods were alternated daily. The fixation device was removed by the nurse every day, and oral care was performed. After oral care, the fixed method was changed, and damaged skin was steered when the tape was pasted (Figs. 4, 5, 6).

### Observation indexes

A patient data collection form was designed, which included the general data of patients, fixation methods, and observation of the catheter displacement, dental condition, and skin and mucosal damage within 14 days after tracheal intubation. The general data included the following: name, age, diagnosis, group, Modified Early Warning Score (MEWS), and Glasgow Coma Scale (GCS). Data were obtained from the medical records of patients and nursing records. The main observation indexes included the displacement and degree of the tracheal catheter, the presence of tooth loosening, and the damage and degree of the facial skin, lip, tongue, and oral mucosa (including rupturing and bleeding).

### Curative effect evaluation standard

In the present study, catheter displacement and severity of skin injury were evaluated using the catheter displacement assessment scale and skin injury severity scale. When the insertion depth of the catheter moves forward or backward by 0.5–0.8 cm and does not cause the catheter to slip down or affect the right lung ventilation, it is defined as catheter displacement. Severe displacement: This occurs when the insertion depth of the catheter moves forward or backward by > 0.8 cm, which causes the catheter to slip down or slip into the right bronchus, affecting the right lung ventilation. All skin and mucosal injuries and erosions associated with the intubation were considered as skin injuries.

### Statistical methods

In the present study, data were analyzed using statistical software SPSS 20.0. Measurement data were expressed as mean ± standard deviation (*x* ± SD). Count data were expressed as a percentage (%). The test of normality was conducted using the Shapiro–Wilk test (W-test). For multiple comparisons, each value was compared by one-way ANOVA following Dunnett tests when each datum conformed to a normal distribution, while the non-normally distributed continuous data were compared using non-parametric tests. Counting data were compared using the basic Chi-square test. *P* < 0.05 was considered statistically significant. Corrected *P*-values were used for multiple comparisons (P/n).

## Results

### General characteristics

A total of 95 patients were enrolled in the present study. According to the order of tracheal intubation after hospitalization, these patients were randomly divided into three groups and observed using the inter-group control method. Three methods were used to fix the tracheal intubation of patients: method 1, the twill tape method; method 2, the fixator method; and method 3, the alternation method. Method 1 was applied in 31 patients, which included ten patients with respiratory failure, ten patients with severe pneumonia, one patient with acute exacerbation of chronic obstructive pulmonary disease (AECOPD), one patient with heart failure, two patients with hemoptysis, two patients with digestive tract hemorrhage, one patient after cardiopulmonary resuscitation, two patients with consciousness disturbance, and two patients with cerebral infarction. The average intubation time was 8.9 ± 2.7 days. Method 2 was applied in 31 patients, which included four patients with respiratory failure, nine patients with severe pneumonia, one patient with AECOPD, four patients with heart failure, two patients with cardiopulmonary resuscitation, three patients with gastrointestinal bleeding, one patient with hemoptysis, one patient with cerebral infarction, four patients with disturbance of consciousness, one patient with renal failure, and one patient with drug poisoning. The average intubation time was 9.5 ± 3.5 days. Method 3 was applied in 33 patients, which included ten patients with respiratory failure, 12 patients with severe pneumonia, one patient with severe pancreatitis, two patients with acute myocardial infarction, two patients with consciousness disturbance, one patient with myasthenia gravis, one patient with brainstem hemorrhage, one patient with intestinal obstruction, one patient with renal failure, one patient with drug poisoning and one patient after resuscitation. The average intubation time was 8.9 ± 3.0 days. The differences in gender, age, MEWS, and GCS score before tracheal intubation among the three groups were not statistically significant (*P* > 0.05, Table 1). Furthermore, there was no significant difference in intubation time among the three groups (*P* > 0.05).

**Table 1:** Comparison of general data before tracheal intubation among the three groups

Items	The twill tape method (*n* = 31)	The fixator method (*n* = 31)	The alternation method (*n* = 33)	Test statistic	*P* value
Male/female	14/17	20/11	22/11	3.644	0.162
Average age (years old)	75.3 ± 10.9	70.1 ± 14.6	67.0 ± 15.7	2.840	0.064
NEWS	5.3 ± 1.9	5.9 ± 1.5	5.3 ± 1.4	1.625	0.203
GCS	3.2 ± 0.5	3.2 ± 0.5	3.2 ± 0.7	0.086	0.918

The differences in general data, such as gender, age, MEWS, and GCS score before tracheal intubation among the three groups were not statistically significant (*P* > 0.05). Hence, these three groups were comparable (Table 1).

### Comparison of facial and lip injuries

Facial and lip injuries were compared among the three methods (*P* = 0.061, Table 2).

**Table 2:** Comparison of facial and lip injuries, tongue injuries, teeth loosening and catheter displacement among three groups

Groups	Facial and lip injuries	Tongue injuries	Teeth loosening	Catheter displacement
The twill tape method	16/31 (51.6%)	14/31 (45.2%)	16/31 (51.6%)	12/31 (38.7%)
The fixator method	18/31 (58.1)	24/31 (77.4)^a^	27/31 (87.1)^a^	11/31 (35.5)^c^
The alternation method	26/33 (78.8)	31/33 (93.9)^b^	30/33 (90.9)^b^	23/33 (69.7)^b^

^a^Compared between the twill tape method and the fixator method, *P* < 0.05

^b^Compared between the twill tape method and the alternation method, *P* < 0.05

^c^Compared between the fixator method and the alternation method, *P* < 0.05

### Comparison of tongue injuries

The difference in tongue injuries among the three methods was statistically significant (*P* < 0.001). In the pairwise comparisons, the difference in tongue injuries between groups 1 and 2 was statistically significant in the Chi-square test (*P* < 0.05). The difference in tongue injuries between groups 1 and 3 was statistically significant in the Chi-square test (*P* < 0.05), but the difference in tongue injuries between groups 2 and 3 was not statistically significant in the Chi-square test (*P* > 0.05) (Table 2).

### Comparison of teeth loosening

The difference in teeth loosening among the three methods was statistically significant (*P* < 0.001). In the pairwise comparisons, the difference in teeth loosening between groups 1 and 2 was statistically significant in the Chi-square test (*P*[1, 2] = 0.002 < 0.05). The difference in teeth loosening between groups 1 and 3 was statistically significant in the Chi-square test (*P* < 0.05), but the difference in teeth loosening between groups 2 and 3 was not statistically significant in the Chi-square test (*P*[2, 3] = 0.625 > 0.05) (Table 2).

### Comparison of catheter displacement

The difference in catheter displacement among the three methods was statistically significant (*P* = 0.013 < 0.05). In the pairwise comparisons, the difference in catheter displacement between groups 1 and 2 was not statistically significant in the Chi-square test (*P*[1, 2] = 0.793 > 0.05). The difference in catheter displacement between groups 1 and 3 was not statistically significant in the Chi-square test (*P*[1, 3] = 0.013 > 0.05), and the difference in catheter displacement between groups 2 and 3 was statistically significant in the Chi-square test (*P*[2, 3] = 0.006 < 0.05) (Table 2).

## Discussion

The results of the present study revealed that the fixator and alternation methods were more effective in protecting the tongue mucosa and teeth. The alternation method was significantly superior to the other two methods in maintaining the position of the endotracheal intubation. The difference in facial and lip injuries between the three methods was not statistically significant. The reason that the difference in facial and lip injuries among the three methods was not statistically significant may be the small sample size. In subsequent studies, these sample sizes should be further expanded. The differences in the incidence of tongue injury and tooth loosening between group 1 and group 2 and 3 were statistically significant. In combination with this and the damage-protected rate, it is suggested that the fixator and alternation methods were more effective in protecting the tongue mucosa and teeth. The difference in the displacement of the tracheal intubation between groups 2 and 3 was statistically significant. In combination with this and the incidence, it is suggested that the alternation method significantly reduces the displacement of the endotracheal intubation. The difference in the displacement of the tracheal intubation between the twill tape method and the fixator method was not statistically significant. This result proves that the alternation method is significantly superior to the other two methods in maintaining the position of the endotracheal intubation. Therefore, the fixator method can significantly reduce intraoral injury, is more suitable for older people with weak tongue mucosa and looser teeth, and can be used as the first choice for intubation patients who are conscious and have resistance to foreign bodies in the oral cavity. Furthermore, the alternation method has significant advantages over the other two methods in tongue protection and teeth protection, and in maintaining the location of the tracheal intubation.

In recent years, scholars have conducted a number of explorations on the fixation methods of orotracheal intubation [9, 10]. In focusing on the fixation effect, more and more attention has been given to the improvement of comfort to make it more convenient, comfortable, and personalized based on a firm fixation [11–14]. The above-mentioned three methods have their own advantages and disadvantages. The advantage of method 1 is the low cost [15]. The disadvantages are possible pressure injuries of the upper part of the tongue and lip caused by the mouth pad and intubation catheter, and injury of the skin around the mouth and face caused by the tape's paste. The mouth pad is 8 cm long, so its placement in the mouth affects swallowing and the discomfort and outflow of oral secretions from the corners of the mouth. Instinctively, the patient’s tongue pushes out the bite block and causes catheter displacement [16, 17]. The advantage of method 2 is the comfort of the skin of the lips and face without the tape's paste. The mouth pad is short and acts as one with the fixator. Furthermore, it is fixed into position and will not affect the swallowing of patients or reduce catheter displacement. Since swallowing is not affected, patients have less oral secretion. However, the disadvantages are possible compression injuries of the apex of the tongue and possible teeth loosening caused by the high hardness and flaky edge of the plastic dental pad [2, 4, 18, 19]. Method 3 reduces the risk of long-term use of any fixation method by alternating the fixation method, thereby reducing the rate of catheter displacement, tongue breakage, and tooth loosening. This effectively reduces the injury caused by endotracheal intubation and protects the organ function of patients [20]. Comprehensively, the alternation method has significant advantages over the other two methods and is worthy of clinical popularization and application.

The present study still has the following limitations. First, the study was a case–control study and not a randomized controlled trial, and the blind method was not used. Therefore, there is still a certain risk of bias. Second, the study was a single-center clinical trial, and the included sample size was small. Hence, multi-center clinical trials with larger sample sizes are still needed. It was an observational study, so there is the possibility of bias in all the groups due to a lack of proper controls. Third, the results indicate that the alternation method is superior to the other two methods in maintaining the position of the endotracheal intubation. However, it is still not clear whether it is purely the effect of the alternation method and should be further researched.

## Conclusion

The fixator method can significantly reduce intraoral injury and is more suitable for older people with weak tongue mucosa and loose teeth, and is worth popularizing among a wider group.

## Data Availability

The datasets used and/or analyzed during the current study available from the corresponding author on reasonable request.

## Abbreviations

ICU: Intensive care unit
EICU: Emergency intensive care unit
MEWS: Modified Early Warning Score
GCS: Glasgow Coma Scale
x ± SD: Mean ± standard deviation
AECOPD: Acute exacerbation of chronic obstructive pulmonary disease
MEWS: Modified Early Warning Score
GCS: Glasgow Coma Scale
